# Diaphragmatic motility assessment in COPD exacerbation, early detection of Non-Invasive Mechanical Ventilation failure: a pilot study

**DOI:** 10.1186/2036-7902-6-S2-A6

**Published:** 2014-08-27

**Authors:** Fabio Giuliano Numis, Lucia Morelli, Giorgio Bosso, Mario Masarone, Sara Cocozza, Anita Costanzo, Fernando Schiraldi

**Affiliations:** 1 UOC Pronto Soccorso-Medicina d’Urgenza, Ospedale S. Paolo, Napoli, Italy; 2 Dipartimento di Medicina Interna ed Epatologia, Università di Salerno, Italy

## Background

Patients with respiratory failure due to Chronic Obstructive Pulmonary Disease (COPD) have decreased diaphragmatic mobility [1]. Non Invasive Mechanical Ventilation (NIMV) is a cornerstone in COPD exacerbation therapy. The availability of early predictors of NIMV failure may be helpful to guide decision-making. Only pH, respiratory rate and PaO2/FiO2 have been considered predictors of response to NIMV [2]. Ultrasonographic (US) assessment of diaphragmatic kinetic is a fast, reliable and reproducible method [3], but its predictive value on NIMV success is not known.

## Objective

Primary endpoint was to evaluate if the diaphragmatic excursion measurement was able to predict a longer weaning time. Secondary endpoint was to find out a cut-off value of diaphragmatic excursion and a weaning time interval able to predict worst outcome.

## Methods

Fifty-two (39 males, aged 71±7 years) Caucasian patients with COPD exacerbation treated with NIMV were enrolled. Diaphragm motility was assessed by ultrasonography before starting ventilation at 6 and 24 hours and at the weaning from NIMV. The diaphragmatic excursion (centimeters); the inspiratory and expiratory times (seconds); the inspiration and expiration velocity (cm/sec), the breathing period (seconds), the diaphragm motion time (seconds) and the diaphragm resting time (seconds) were evaluated.

## Results

Forty-five patients completed the study. The mean time on NIMV was 4.11 ± 1.07 days, with a total time of ventilation of 32.6 ± 86 hours. All the ultrasound diaphragm motility parameters, except for inspiration and expiration velocity, significantly improved at the weaning. Diaphragm excursion at the baseline was significantly correlated with pH (r=0.458; p=0.002), PaO_2_/FiO_2_ (r=0.567; p<0.001), and weaning time (r=0.774; p<0.0001). In a multiple linear regression analysis only diaphragm excursion was significantly associated with longer weaning time (coefficient of estimated model -9.247; Standard Error 3.101; p=0.003). ROC curve with weaning time longer than 36 hours was considered as positive state. The AUC value was 0.912 (Standard Error 0.015, p<0.001). The higher sensitivity rate (100%) was achieved with a specificity rate of 86.7% and a cut-off value of 3.165 cm, therefore patients with an excursion lower than 3.165 cm should be weaned after at least 36 hours, while patients with an excursion higher than 3.165 cm could be weaned within 36 hours.

## Conclusion

US measurements of diaphragmatic performance may have a role in the early evaluation of exacerbation of COPD and in predicting the response to NIMV therapy, it should be included as a routine test in patients presenting to ED with ECOPD.
